# Characterization and Application in Natural Rubber of *Leucaena* Leaf and Its Extracted Products

**DOI:** 10.3390/polym15183698

**Published:** 2023-09-08

**Authors:** Pattamaporn Klongklaew, Phimthong Khamjapo, Pongdhorn Sae-Oui, Pairote Jittham, Surapich Loykulnant, Weenusarin Intiya

**Affiliations:** National Metal and Materials Technology Center (MTEC), National Science and Technology Development Agency (NSTDA), Klong Nueng, Khlong Luang 12120, Pathum Thani, Thailand; pattamaporn.klo@ncr.nstda.or.th (P.K.); eraciab19@gmail.com (P.K.); pongdhor@mtec.or.th (P.S.-O.); pairotej@mtec.or.th (P.J.); surapicl@mtec.or.th (S.L.)

**Keywords:** *Leucaena*, vulcanization, mechanical properties, eco-friendly, additives, protein, rubber

## Abstract

*Leucaena* is a fast-growing tree in the legume family. Its leaf contains a significant amount of protein and is thus widely used as fodder for cattle. To broaden its application in the rubber field, the effects of *Leucaena* leaf powder and its extracted products on the cure characteristics and mechanical properties of natural rubber were investigated. The extraction of *Leucaena* leaf was carried out by using a proteolytic enzyme at 60 °C. The digested protein was separated from the residue by centrifugation. Both digested protein and residue were then dried and ground into powder, namely digested protein powder and residual powder, respectively, before being characterized by Fourier transform infrared spectroscopy, scanning electron microscope, thermogravimetric analysis, X-ray diffraction, particle size determination, and protein analysis. After being added to natural rubber at 3 parts per hundred rubber, they significantly reduced both the scorch time and the optimum cure time of the rubber compounds, probably due to the presence of nitrogen-containing substances, without a significant sacrifice of the mechanical properties. For instance, the optimum cure time decreased by approximately 25.5, 35.4, and 54.9% for *Leucaena* leaf powder, residual powder, and digested protein powder, respectively. Thus, they can be used as green and sustainable fillers with a cure-activation effect in rubber compounding.

## 1. Introduction

Natural rubber (NR) is a biopolymer obtained from the para rubber tree (*Hevea braziliensis*), consisting of isoprene units (C_5_H_8_) that are linked together to form long polymer chains, namely cis-1,4-polyisoprene, with a trace of other organic impurities such as proteins and phospholipids. The glass transition temperature (T_g_) of NR is approximately −70 °C. Due to its exceptionally high elasticity, high strength, and good resistance to many corrosive substances, NR is extensively used in many applications, including gloves, rubber bands, threads, condoms, etc. However, the abundance of double bonds in isoprene units makes NR highly susceptible to degradation by heat, oxygen, and ozone. The addition of antidegradants is thus inevitable. Without crosslinking, the elasticity and mechanical properties of NR are not sufficiently high for practical applications. To obtain certain desirable properties, many additives such as fillers, sulfur, activators, and accelerators are added to rubber during the manufacturing process to reinforce or permit sulfur vulcanization [1]. Vulcanization is a process in which individual rubber molecules are linked together, forming a three-dimensional network of interconnected chains through chemical crosslinks. For unsaturated rubbers such as NR, vulcanization generally occurs in the presence of sulfur and heat. However, vulcanization by sulfur alone is an extremely slow and uneconomical process. The addition of accelerators in conjunction with activators is, therefore, inevitable to increase the vulcanization speed and permit vulcanization to proceed at a lower temperature and with greater efficiency. They achieve this by reacting with the sulfur to form a reactive intermediate called a sulfurating agent. This, in turn, reacts with cure sites (allylic hydrogen atoms) in the rubber to bring about vulcanization. Accelerators are also classified as primary or secondary accelerators based on their role in a given compound. Unfortunately, some of these rubber-compounding ingredients are considered toxic to the environment and humans. For instance, zinc oxide (ZnO), the most widely used inorganic activator for sulfur vulcanization, is known to be hazardous for aquatic ecosystems [2,3]. For humans, toxicity from rubber chemicals is mostly focused on vulcanization accelerators. Many types of accelerators have been reported to cause allergies or release carcinogenic nitrosamines; for instance, some of the accelerators in the thiuram group, such as tetramethylthiuram monosulfide (TMTM) and tetramethylthiuram disulfide (TMTD) [4]. To reduce the toxicity from nitrosamine, alternative accelerators from natural resources are, therefore, of great interest.

The environmental problems and toxicity caused by rubber-compounding ingredients have recently gained much attention. Chemicals or fillers from natural resources, alternatively called green chemicals or green fillers, are encouraged to replace some additives in rubber production processes due to their eco-friendliness, low toxicity, and good biodegradability. A green composite can be prepared when one or more of the main components come from natural resources [5]. Nowadays, many published works have reported the use of renewable biofillers in rubber composites with the intention of enhancing strength, improving biodegradability or a combination of both. Recently, trypsin hydrolyzed gliadin (THGd) from wheat protein has been reported as a reinforcing biofiller in rubber compounds [6]. Potato starch nanocrystals, obtained from potato starch granules by sulfuric acid hydrolysis, were added to NR to increase the biodegradability of rubber products [7]. Apart from starch, cellulose has also gained much attention from many rubber technologists around the globe because it is abundant, easily available, and low-cost. Most importantly, cellulose can be extracted from a variety of plants and can be prepared in various sizes, ranging from micro-scale celluloses (e.g., microfibers) to nano-scale celluloses (e.g., cellulose nanofibrils, cellulose nanocrystals, nanocellulose whiskers, etc.) [8,9,10,11,12,13]. Despite its high hydrophilicity, surface treatment with bifunctional compounds such as silane coupling agents can be applied to improve its compatibility and thus enhance the magnitude of rubber-filler interaction with many non-polar rubbers such as natural rubber (NR), butadiene rubber (BR), and styrene butadiene rubber (SBR) [14,15,16,17]. The feasibility of using household or industrial wastes, particularly spent coffee ground [18], lignin-silica material (LSM) from rice husk [19], rice husk ash [20,21], fly ash [22], waste eggshell powder [23], as a filler in rubber has also been reported. In the tire industry, great attention has also been paid to the replacement of toxic rubber processing oils, particularly distillate aromatic extract (DAE) oil, which contains a high quantity of carcinogenic polycyclic aromatic hydrocarbons (PAHs), with less toxic mineral oils such as treated distillate aromatic extract (TDAE) oil or with non-toxic vegetable oils such as palm oil [24,25].

According to the literature, many biomaterials have been employed as additives in rubber compounding with the intention of replacing fillers and toxic plasticizers. So far, various forms of cellulose have been extracted from plants and used as a biofiller in rubber. Surprisingly, little attention has been paid to the direct application of plant leaves, which contain other components such as chlorophyll and proteins, in rubber compounding. It is, therefore, interesting to study the effect of plant leaves on various properties of rubber. In this study, *Leucaena* leaf was selected as a potential green material that can act as a biofiller and, in the meantime, significantly reduce the vulcanization time of the rubber because it contains a significant amount of protein (20–27%) [26] and other nitrogen-containing compounds. It is also easily available because *Leucaena* is known as a fast-growing tree in the legume family that is capable of fixing nitrogen from the air. It grows well against all plants, survives in all soil conditions, and is resistant to diseases and insects. In addition, it can reproduce quickly due to the large number of pods and seeds, coupled with a high germination rate. Hence, *Leucaena* can often be seen in various parts of the world. The *Leucaena* leaf is also commercially available in powder form because it is produced as fodder for cattle.

This work is intended to investigate the effects of *Leucaena* leaf powder (LLP) and its extracted products on the cure characteristics and mechanical properties of NR. *Leucaena* leaf was initially dried, ground, and sieved prior to being used. Protein was also extracted from LLP by using alkali and proteolytic enzymes. Both digested protein and residue were dried and ground into powder, called digested protein powder (DPP) and residual powder (RP), respectively, before being incorporated into NR.

## 2. Experimental

### 2.1. Materials

The *Leucaena* leaves were obtained from Hang Dong, Chiang Mai, Thailand. Natural rubber (NR; STR 5L) was purchased from Union Rubber Products Corp., Ltd. (Bangkok, Thailand). Deionized water was received from a water purification system (Thermo Scientific, Langenselbold, Germany). The other chemicals used in the present work are tabulated in Table 1.

### 2.2. Preparation of LLP, DPP, and RP

**Figure 1:**
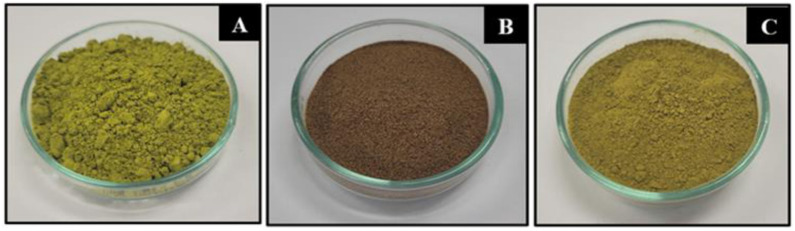
Photographs of (**A**) LLP, (**B**) DPP, and (**C**) RP.

The fresh *Leucaena* leaves were dried in an oven at 105 °C for 2 h to remove the moisture. The petiole and midrib were separated and discarded. Dried *Leucaena* leaves were ground by an automatic grinder (Hok Tai Machinery, Muang, Samutprakan, Thailand) for 10 min and sieved through a 40-mesh screen to obtain the fine green powder, called LLP, as shown in Figure 1A. Protein extraction of LLP was carried out using procedures adapted from the literature [27,28,29]. Firstly, LLP (20.0 g) was dispersed in 0.1 mol/L NaOH, and the pH of the mixture was adjusted to 10.0. After stirring at 1000 rpm for 60 min at room temperature, the pH was adjusted to 8.0 by using 1.0 mol/l HCl. The mixture’s temperature was then raised to 60 °C, the optimal temperature for proteolytic enzyme activity, before adding 5.0 mL of proteolytic enzyme (0.7% *v*/*v*). The mixture was stirred for 60 min. To restrain the enzyme activity, the mixture was heated up to 95 °C and stirred for 15 min. The mixture was kept at room temperature to cool down and subsequently centrifuged at 9000 rpm at 4 °C for 10 min. After centrifugation, the mixture was separated into two parts, i.e., brown supernatant (digested proteins) and green precipitate (residual solid). The brown supernatant was dried in an oven at 40 °C for 24 h, ground by mortar, and sieved through a 40-mesh screen to obtain the digested protein powder (DPP), as shown in Figure 1B. The precipitate was washed with DI water four times before being dried at 60 °C for 12 h, ground by an automatic grinder, and sieved through a 40-mesh screen to obtain the residual powder (RP), as shown in Figure 1C.

### 2.3. Characterization of LLP, DPP, and RP

The functional groups of LLP, DPP, and RP were studied by a Fourier transform infrared (FT-IR) spectrophotometer (Bruker Tensor 27, Ettlingen, Germany) at a resolution of 4 cm^−1^ and scanning range of 4000–400 cm^−1^. The samples were thoroughly mixed with potassium bromide (KBr) and shaped into a disc prior to the analysis. The thermal stability was investigated by a thermogravimetric analyzer (TGA: Mettler, Toledo, Melbourne, Australia). The test was carried out under a nitrogen atmosphere from 30 to 600 °C at a heating rate of 10 K/min. The crystalline structure of the samples was investigated by an X-ray diffractometer (JEOL JEX-3530, Tokyo, Japan). The surface morphology was studied by a field emission scanning electron microscope (FE-SEM SU5000, Hitachi High-Tech, Tokyo, Japan). The particle size of the samples was studied by Mastersizer-S (Malvern, Worcestershire, UK). The determination of protein content was carried out using the Lowry method [30].

### 2.4. Rubber Compound Preparation and Testing

The rubber formulas used in this study are given in Table 2. Mixing was performed in an internal mixer (Polylab OS, Thermo Scientific, Germany) under the following conditions: an initial temperature of 60 °C, a rotor rotation speed of 40 rpm, and a fill factor of 0.8. NR was initially masticated for 2 min before adding zinc oxide and stearic acid. After mixing for 2 min, the prepared green powder (LLP, DPP, or RP) was added and mixed for another 2 min before adding TBBS and sulfur. Mixing was completed at the 10th minute. The rubber compounds were removed from the internal mixer, sheeted on a two-roll mill, and left at room temperature overnight before being tested.

The cure characteristics of the rubber compounds, including scorch time (t_s1_), optimum cure time (t_c_95), maximum torque (M_H_), and minimum torque (M_L_), were measured using a moving die rheometer (MDR TechPRO MD+, CG Engineering Co., Ltd., Sam Khok, Thailand) at 150 °C for 30 min based on ISO 6502-3 [31]. After the cure characteristic test, the rubber compounds were compression molded in a hydraulic hot press at 150 °C using the pre-determined optimum cure time to prepare vulcanized rubber sheets with approximately 2 mm thickness. The mechanical properties of the vulcanized rubbers were then investigated. The hardness test was carried out using a Wallace Shore A durometer based on ISO 48-4 [32]. 100% modulus, elongation at break, and tensile strength were evaluated using a universal testing machine (NRI-TS500-20B (Extra), Narin Instrument Co., Ltd., Pak Nam, Thailand) based on ISO 37 [33] (Die Type 2). Crosslink density was determined by immersing the weighed rubber specimens in toluene at room temperature for 7 days. The swollen specimens were then blotted with filter paper and immediately weighed. The swelling ratio was calculated and used to indirectly represent the magnitude of crosslink density. The morphology of the vulcanizates was studied by using an optical microscope (Axioskop, ZEISS Microscope, Oberkochen, Germany).

## 3. Results and Discussion

### 3.1. Basic Characterization of LLP, DPP, and RP

#### 3.1.1. Fourier Transform Infrared Spectroscopy (FTIR)

**Figure 2:**
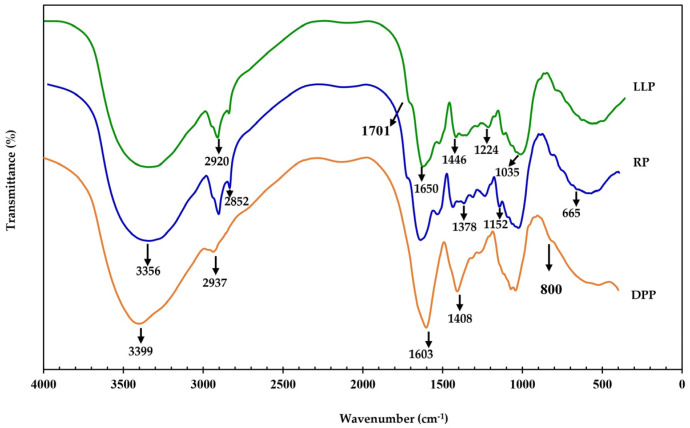
The FTIR spectra of LLP, DPP, and RP.

The functional groups of LLP, DPP, and RP were investigated by FTIR spectroscopy, and the results are given in Figure 2. As DPP and RP were extracted from LLP, the FTIR spectrum of LLP showed the characteristic peaks of both DPP and RP. In the DPP spectrum, the characteristic peaks of protein are quite obvious, i.e., the peak at 3399 cm^−1^ is attributed to the vibration of N-H group; the peak at 2937 cm^−1^ is related to the C-H stretching vibration; the strong peak at 1603 cm^−1^ is related to the vibration of C=O in amide groups of protein and the peaks at 1408 cm^−1^ and 800 cm^−1^ are assigned to the vibrations of C-N, and O=C-N, respectively. Similar results are also reported in the literature [34,35].

In the RP spectrum, the characteristic peaks of cellulose are found, i.e., the peak at 3356 cm^−1^ corresponding to the stretching vibration of O-H bonds, including inter- and intra-molecular H-bond vibrations in cellulose, the peaks at 2920 cm^−1^ and 2852 cm^−1^ belonging to the stretching vibration of C-H bonds, the peak at 1446 cm^−1^ corresponding to the CH_2_ intertwined in the cellulosic material, the peak at 1378 cm^−1^ belonging to the C-H bending vibration, the strong peak at 1152 cm^−1^ assigned to the stretching vibration of C-O-C bonds, and the small peak at 899 cm^−1^ associated with the C-O-C stretching vibration of cellulosic *β*-glycosidic linkages [13,36,37,38]. Apart from the characteristic peaks of cellulose, the spectrum of RP showed the characteristic peaks of chlorophyll, i.e., the small peak at 1701 cm^−1^ belonging to the absorption of C=O of normal ketone, the peaks at 1650 cm^−1^ attributed to the stretching vibrations of C=C bonds of alkene and C=O bonds of ketone in which the oxygen is coordinated with magnesium, the peak at 1446 cm^−1^ related to the C=C stretching in the aromatic ring, the peak at 1300 cm^−1^ attributed to the vibration of C-N bonds of the tetrapyrrole ring in chlorophyll, and the peak at 1224 cm^−1^ related to the C-O stretching vibration of ester groups [39,40]. The results clearly revealed that the main components of RP were cellulose and chlorophyll. The presence of chlorophyll explains why the color of the RP was dark green (see Figure 1C). The spectrum of LLP showed a large number of peaks because it contains chlorophyll, protein, and cellulose [41,42,43].

#### 3.1.2. Thermogravimetric Analysis (TGA)

**Figure 3:**
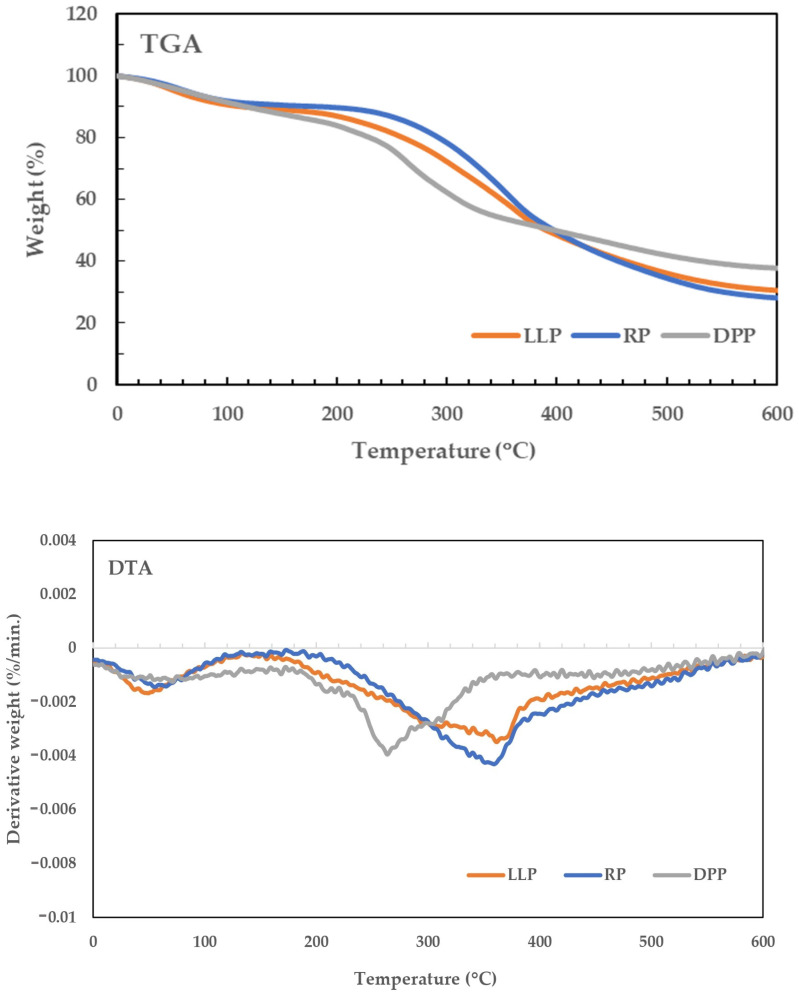
TGA/DTG curves of LLP, RP, and DPP.

The TGA/DTG curves of LLP, DPP, and RP are illustrated in Figure 3. For DPP, multiple decomposition stages were found. The first stage took place below 100 °C due to the elimination of free and bonded water [44]. The second stage of mass loss, which occurred at approximately 200–360 °C, was attributed to the cleavage of the covalent peptide bonds. The last stage of mass loss occurred slowly above 360 °C because of the breakage of S-S, O-N, and O-O linkages from protein molecules [45,46,47]. For RP, almost 10% mass loss was found in the early stage of the pyrolysis at temperatures up to 100 °C, which can be explained by the dehydration of moisture adsorbed on the cellulose surface. The mass loss found at temperatures above 200 °C is attributed to the decomposition of both cellulose and chlorophyll. From the literature, both cellulose and chlorophyll have a very similar decomposition pattern with a broad decomposition temperature in the range of 200–500 °C. For cellulose containing both amorphous and crystalline regions, the decomposition might be separated into two steps, i.e., the first step taking place at lower temperatures, say 200–360 °C, is attributed to the decomposition of the amorphous cellulose, while the second one generally found at higher temperatures is the consequence of the decomposition of the crystalline cellulose [12]. Chlorophyll also has two decomposition stages. The first stage involves the decomposition of ester linkages releasing phytyl (3,7,11,15-tetramethyl-2-hexadecenyl) chains at temperatures 200–340 °C, whereas the second stage found at higher temperatures refers to the decomposition of the skeleton of the isoprenoid chain [48]. It can be observed that the decomposition curve of LLP is very similar to that of RP, but a slightly greater mass loss was found at temperatures between 200 and 360 °C, possibly due to the additional loss of protein in LLP. At the end of the test, a significant amount of residue, containing mainly carbonaceous substance (biochar), was observed in all samples, i.e., approximately 29% for LLP, 37% for DPP, and 27% for RP, which could be explained by the presence of nitrogen-containing substances (proteins and chlorophyll) in the samples. DPP showed the highest content of biochar because it is a protein-rich sample containing a large number of nitrogen atoms in amino acids.

#### 3.1.3. X-ray Diffraction Spectroscopy (XRD)

**Figure 4:**
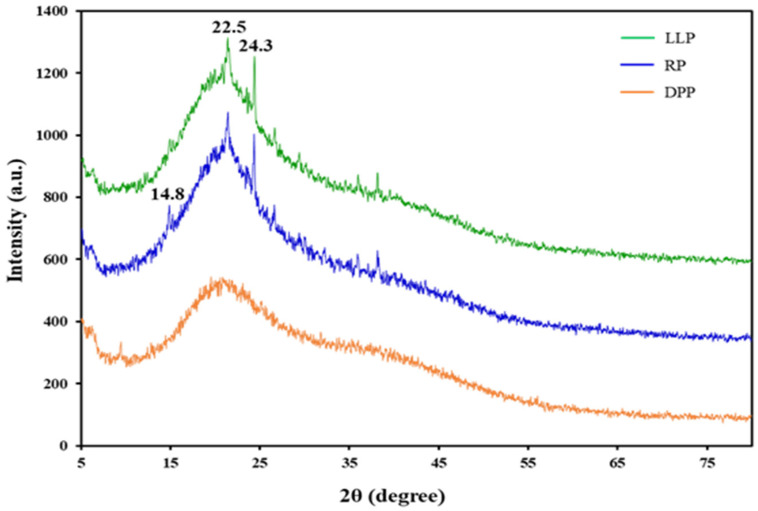
The XRD diffraction patterns of LLP, DPP, and RP.

X-ray diffraction (XRD) patterns of LLP, DPP, and RP are shown in Figure 4. The diffractogram of DPP did not show any sharp peaks due to the amorphous nature of the protein. A similar observation has been reported [49]. The XRD patterns of LLP and RP showed broad amorphous peaks with some tiny sharp peaks at 2θ of 14.8°, 22.5°, and 24.3°, indicating that the cellulose and other substances in LLP and RP are mostly in amorphous forms.

#### 3.1.4. Scanning Electron Microscope (SEM)

**Figure 5:**
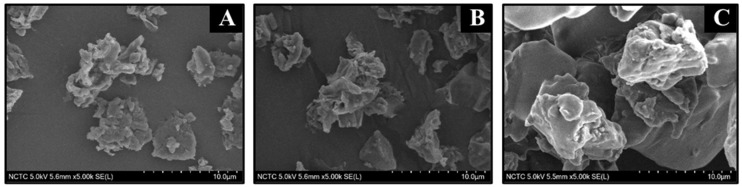
The SEM images of (**A**) LLP, (**B**) RP, and (**C**) DPP.

The surface morphology of LLP, DPP, and RP was studied by SEM, and the SEM images are given in Figure 5. Apparently, all samples exhibited an irregular shape with relatively large particle size, ranging from 10 to 70 µm. It can be observed that DPP has a slightly larger particle size than LLP and RP, possibly due to the difference in grinding technique used during the sample preparation, i.e., DPP was ground by mortar, while LLP and RP were ground by an automatic grinding machine.

#### 3.1.5. Particle Size

Measurement of particle size was carried out by the Mastersizer. As DPP was highly soluble in water, its particle size could not be measured. The average particle size of LLP was approximately 31.1 ± 0.3 µm. The average particle size was significantly reduced when protein was extracted from the LLP particles. In this study, the average particle size of the RP was about 23.5 ± 1.2 µm.

#### 3.1.6. Protein Analysis

The protein contents of LLP, DPP, and RP were investigated by the Lowry method, and the results are tabulated in Table 3. The protein content of LLP was 21.3%, which is slightly lower than the values recently reported in the literature [50], which revealed that the protein content of *Leucaena* leaf was in the range of 22.8–29.9%, depending on the environmental field, species, and season. As expected, DPP had the highest protein content (approximately 49%). RP, on the other hand, had the lowest protein content (approximately 7%) because most proteins were hydrolyzed and extracted from LLP by NaOH and enzyme. The results revealed that the protein extraction under the conditions used herein was not completed because there was still some protein left in RP.

### 3.2. Properties of Rubber Compounds

#### 3.2.1. Cure Characteristics

**Figure 6:**
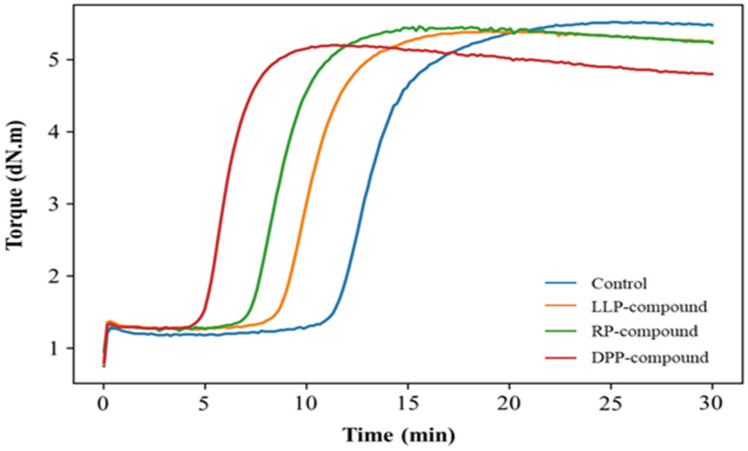
The cure curves of the rubber compound without the green powder (control) and the rubber compounds with 3 phr of LLP, DPP, and RP.

The cure curves of the rubber compound without the green powder (control) and those with 3 phr of LLP, DPP, and RP are displayed in Figure 6. The values of scorch time (t_s1_), optimum cure time (t_c_95), minimum torque (M_L_), and maximum torque (M_H_) are also tabulated in Table 4. Without the addition of the green powder, the rubber compound (control) had a very long scorch time (t_s1_ = 11.45 min) and an optimum cure time (t_c_95 = 17.14 min). The cure curve noticeably shifted to the left when LLP, DPP, and RP were added, indicating the capability to reduce the onset of the sulfur vulcanization reaction of the green powder. The greatest shift was found in DPP, followed by RP and LLP, respectively. The results revealed the highest efficacy of DPP in reducing the scorch time and cure time of the rubber compounds. This may be attributed to the fact that DPP had a relatively high protein content (~49%), which could accelerate the sulfur vulcanization of the isoprene rubber, according to the literature [51,52,53,54]. It has recently been reported that proteins decrease the activation energy of the crosslinking process through the formation of coordination interactions between Zn^2+^ and amide bonds of proteins, which improves the solubility of Zn^2+^ in the matrix and further contributes to the crosslinking reaction [55]. The resultant soluble Zn^2+^ then reacts with 2-mercaptobenzothiazole (MBT), a by-product from the radical cleavage of TBBS, to produce the zinc salt of 2-mercaptobenzothiazole (ZnMBT). The ZnMBT further reacts with sulfur, forming a complex referred to as a ZnMBT-polysulfide. This complex finally reacts with NR molecules at reactive cure sites to form a crosslinking precursor with benzothiazole-group terminated polysulfides linking to the rubber. The polysulfide contained in the crosslink precursor can be radically cleaved, and the resultant sulfur radicals can recombine with the carbon radicals of the rubber chains to form crosslinks [56].

Surprisingly, RP showed greater efficacy than LLP in terms of cure time reduction, despite its lowest protein content. The results indicated that there are other active substances in RP that can activate the vulcanization reaction. Attention is given to the nitrogen-containing substances in the RP, especially chlorophyll, which can be clearly seen from its dark green color. Even though the vulcanization reactions in the accelerated system are very complicated and not well declared, it is thought that the reduction in vulcanization onset occurs due to the charge transfer between Mg^2+^ in chlorophyll and Zn^2+^ in ZnO, which could improve the solubility of ZnO in the rubber matrix [57]. Compared with DPP and RP, LLP showed a smaller shift in the cure curve. It should be noted that the reduction in vulcanization time found in the RP and LLP systems may partially arise from their high moisture content (up to 10% from the TGA results) because moisture is able to promote the hydrolysis of the sulfenamide accelerators [58,59]. The results in Figure 6 also show the tendency for cure reversion, a decrease in torque after full cure due to non-oxidative thermal aging, in the rubber compounds containing the green powder. Consequently, the utilization of the green powder should be done with great care, i.e., excessive overcuring must be avoided to prevent the deterioration of rubber properties.

It could also be observed that M_L_ increased slightly in the presence of LLP, DPP, and RP. This is understandable because the solid particles of LLP, DPP, and RP can act as fillers in the rubber matrix, leading to a small increase in compound viscosity. Apparently, a slight reduction in M_H_ was observed in the rubber compounds incorporated with the green powder, despite the reduction in scorch and cure times indicating a slight decrease in cure state. This can be seen from the swelling test results. It is a fact that the swelling ratio is inversely proportional to the crosslink density of rubber. The swelling test results in Table 4 reveal that the addition of the green powder slightly reduced the crosslink density of the rubber vulcanizates. The swelling test results are in good agreement with the rheometric results.

#### 3.2.2. Mechanical Properties

**Figure 7:**
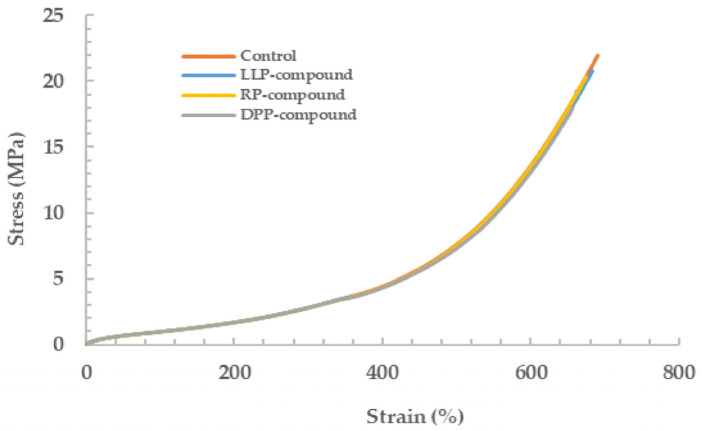
Examples of stress-strain curves of the rubber vulcanizates.

The mechanical properties of the rubber vulcanizates, including hardness, tensile strength, 100% modulus, and elongation at break, are demonstrated in Table 5. Examples of stress-strain curves of the vulcanizates are also displayed in Figure 7.

**Figure 8:**
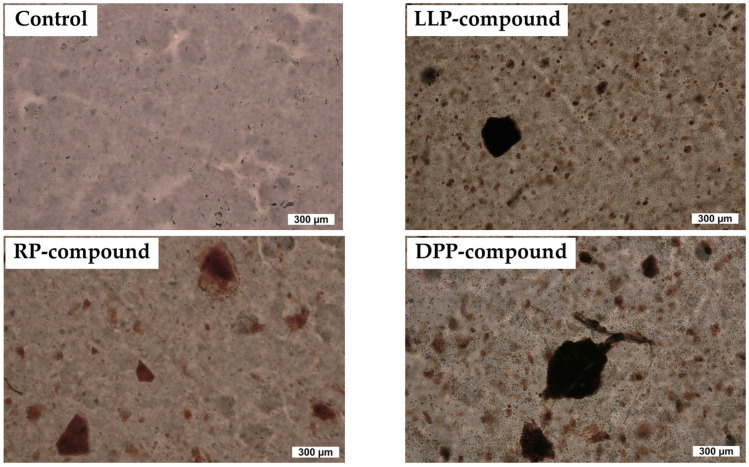
OM images of the rubber compounds.

Without the addition of the green powder, the hardness and 100% modulus of the rubber vulcanizates were approximately 35 Shore A and 0.7 MPa, respectively. Despite the remarkable reduction in cure time in the presence of the green powder samples (LLP, DPP, and RP), both the hardness and 100% modulus of the rubber vulcanizates were hardly affected. Slight decreases in hardness (1 Shore A) and 100% modulus (0.02 MPa) were observed in the presence of the green powder, which is thought to arise from a slight decrease in crosslink density as evidenced by the swelling ratio results (see Table 4). Although the green powder can behave as an inert filler that can increase hardness and 100% modulus due to the hydrodynamic effect, its particle size is too large, and the content is too low to override the effect of crosslink density reduction. Similar results were also found for tensile strength, i.e., the average tensile strengths of all vulcanizates were not significantly different, falling in a narrow range of 20–22 MPa. Theoretically, all green powder samples (LLP, DDP, and RP) contain various quantities of hydrophilic cellulose and proteins that are not compatible with non-polar NR. The addition of these green powder samples to NR should, therefore, deteriorate the tensile strength of the vulcanizates due to poor rubber-filler interaction. In addition, the large particle size of the green powder can induce an area of localized stress, which can contribute to the strength reduction. However, the change in tensile strength was not pronounced in this study because all these powder samples were added at a very low content. Despite the reduction in crosslink density, the elongation at break was found to decrease slightly in the presence of the green powder. Again, the large particles of the green powder embedded in the rubber matrix (see Figure 8) are responsible for such a decrease because they could act as flaws in the rubber matrix.

## 4. Conclusions

The results revealed that LLP could significantly reduce both the scorch and cure times of the rubber compounds without sacrificing the mechanical properties of the vulcanizate. The cure time reduction provided by LLP was expected to arise from the existence of proteins and chlorophyll in LLP, which could activate sulfur vulcanization. As expected, DPP could even further shift the cure curve to the left as compared with LLP, indicating its higher efficacy in reducing the scorch and cure times of the rubber compounds. The cure acceleration of proteins, or amino acids, is well documented. Surprisingly, RP could also reduce both scorch and cure times to a greater extent than LLP, as can be seen from the further shift of the cure curve to the left, despite its lowest protein content. The cure activation found in RP is thought to result mainly from the presence of chlorophyll in RP. Interestingly, the addition of LLP, DPP, and RP to NR significantly reduces both scorch and cure times without a significant sacrifice of the mechanical properties of the rubber vulcanizates. Taken as a whole, both LLP and its extracted products can be used as an eco-friendly filler with a catalytic effect on the onset of sulfur vulcanization of natural rubber.

## Figures and Tables

**Table 1:** Chemicals and their suppliers.

Chemicals	Supplier
Sodium hydroxide (NaOH)	Merck (Darmstadt, Germany)
Proteolytic enzyme (KP3939)	Kao (Tokyo, Japan)
Hydrochloric acid (HCl)	SPS Lab Co., Ltd. (Nonthaburi, Thailand)
Zinc oxide (ZnO)	Thai-Lysaght Co., Ltd. (Ayutthaya, Thailand)
Stearic acid	Kij Paiboon Chemical LP (Bangkok, Thailand)
N-tert-butyl-2-benzothiazylsulfenamide (TBBS)	Monflex Pte. Ltd. (Singapore)
Sulfur	Siam Chemical Industry Co., Ltd. (Muang, Samutprakarn, Thailand)

**Table 2:** The rubber compound formulas.

Ingredients	Control	LLP-Compound	DPP-Compound	RP-Compound
Natural rubber (STR 5 L)	100.0	100.0	100.0	100.0
Zinc oxide	1.0	1.0	1.0	1.0
Stearic acid	1.0	1.0	1.0	1.0
LLP	-	3.0	-	-
DPP	-	-	3.0	-
RP	-	-	-	3.0
TBBS	1.0	1.0	1.0	1.0
Sulfur	1.0	1.0	1.0	1.0

**Table 3:** The protein contents of LLP, DPP, and RP.

Sample	Protein Content (%*w*/*w*)
LLP	21.3
DPP	49.0
RP	7.3

**Table 4:** Cure characteristics and swelling ratio of the rubber compounds.

Compound	t_s1_ (min)	t_c_95 (min)	M_L_ (dN.m)	M_H_ (dN.m)	Swelling Ratio (%)
Control	11.45	17.14	1.16	5.53	411.0 ± 1.3
LLP-compound	7.84	11.87	1.24	5.31	427.6 ± 0.1
DPP-compound	4.75	7.56	1.22	5.05	430.9 ± 0.9
RP-compound	6.72	10.25	1.24	5.19	425.7 ± 0.2

**Table 5:** Mechanical properties of the rubber vulcanizates.

Sample	Mechanical Properties			
	Hardness (Shore A)	Tensile Strength (MPa)	100% Modulus (MPa)	Elongation at Break (%)
Control	34.8 ± 0.2	22.0 ± 1.1	0.69 ± 0.01	707 ± 19
LLP-compound	34.1 ± 0.3	20.3 ± 1.6	0.67 ± 0.02	680 ± 7
RP-compound	33.9 ± 0.4	21.5 ± 0.6	0.67 ± 0.01	685 ± 19
DPP-compound	34.2 ± 0.1	19.6 ± 1.5	0.67 ± 0.01	677 ± 16

## Data Availability

The data presented in this study are available on request from the corresponding author.

## References

[1] Sethulekshmi A.S., Saritha A., Joseph K. (2022). A comprehensive review on the recent advancements in natural rubber nanocomposites. Int. J. Biol. Macromol..

[2] Shinde S.A., More P.R., Ingle A.P., Ingle A.P. (2023). 16—Hazardous effects of nanomaterials on aquatic life. Nanotechnology in Agriculture and Agroecosystems.

[3] Alam M.D., Kumar V., Park S.S. (2022). Advances in Rubber Compounds Using ZnO and MgO as Co-Cure Activators. Polymers.

[4] Alam M.N., Debnath S.C., Boondamnoen O., Kumar K.N., Kim J.H., Choi J. (2022). Synergistic combination of 2-mercaptobenzothiazole (MBT) and nitrosoamine-safe thiuram disulfide as advanced rubber vulcanizing accelerators. J. Elastom. Plast..

[5] Masłowski M., Miedzianowska J., Strzelec K. (2017). Natural rubber biocomposites containing corn, barley and wheat straw. Polym. Test..

[6] DeButts B.L., Thompson R.V., Barone J.R. (2019). Hydrolyzed wheat protein as a self-assembled reinforcing filler in synthetic isoprene rubber vulcanizates. Ind. Crops Prod..

[7] Rajisha K.R., Maria H.J., Pothan L.A., Ahmad Z., Thomas S. (2014). Preparation and characterization of potato starch nanocrystal reinforced natural rubber nanocomposites. Int. J. Biol. Macromol..

[8] Thomas S.K., Dileep P., Begum P.M.S. (2022). Green polymer nanocomposites based on natural rubber and nanocellulose whiskers from Acacia caesia: Mechanical, thermal, and diffusion properties. Mater. Today Proc..

[9] Hirase R., Nagatani A., Yuguchi Y. (2020). Development of powdering method for cellulose nanofibers assisted by zinc oxide for compounding reinforced natural rubber composite. CRGSC.

[10] Lorwanishpaisarn N., Sae-Oui P., Amnuaypanich S., Siriwong C. (2023). Fabrication of untreated and silane-treated carboxylated cellulose nanocrystals and their reinforcement in natural rubber biocomposites. Sci. Rep..

[11] Somseemee O., Sae-Oui P., Siriwong C. (2022). Bio-based epoxidized natural rubber/chitosan/cellulose nanocrystal composites for enhancing mechanical properties, self-healing behavior and triboelectric nanogenerator performance. Cellulose.

[12] Somseemee O., Saeoui P., Schevenels F.T., Siriwong C. (2022). Enhanced interfacial interaction between modified cellulose nanocrystals and epoxidized natural rubber via ultraviolet irradiation. Sci. Rep..

[13] Somseemee O., Sae-Oui P., Siriwong C. (2021). Reinforcement of surface-modified cellulose nanofibrils extracted from Napier grass stem in natural rubber composites. Ind. Crops Prod..

[14] Ren H., Qu Y., Zhao S. (2006). Reinforcement of Styrene-Butadiene Rubber with Silica Modified by Silane Coupling Agents: Experimental and Theoretical Chemistry Study. Chin. J. Chem. Eng..

[15] Jantachum P., Khumpaitool B., Utara S. (2023). Effect of silane coupling agent and cellulose nanocrystals loading on the properties of acrylonitrile butadiene rubber/natural rubber nanocomposites. Ind. Crops Prod..

[16] Zheng J., Han D., Ye X., Wu X., Wu Y., Wang Y., Zhang L. (2018). Chemical and physical interaction between silane coupling agent with long arms and silica and its effect on silica/natural rubber composites. Polymer.

[17] Feng Y., Wang Q., Li L., Ma Y., Li X. (2023). Multiscale analysis of silane coupling agent modified rubber-fiber concrete interfaces. Mater. Today Commun..

[18] Tapangnoi P., Sae-Oui P., Naebpetch W., Siriwong C. (2022). Preparation of purified spent coffee ground and its reinforcement in natural rubber composite. Arab. J. Chem..

[19] Barana D., Orlandi M., Salanti A., Castellani L., Hanel T., Zoia L. (2019). Simultaneous synthesis of cellulose nanocrystals and a lignin-silica biofiller from rice husk: Application for elastomeric compounds. Ind. Crops Prod..

[20] Sae-Oui P., Rakdee C., Thanmathorn P. (2022). Use of rice husk ash as filler in natural rubber vulcanizates: In comparison with other commercial fillers. J. Appl. Polym. Sci..

[21] Dominic M., Joseph R., Sabura Begum P.M., Kanoth B.P., Chandra J., Thomas S. (2020). Green tire technology: Effect of rice husk derived nanocellulose (RHNC) in replacing carbon black (CB) in natural rubber (NR) compounding. Carbohydr. Polym..

[22] Krainoi A., Sripornsawat B., Toh-ae P., Kitisavetjit W., Pittayavinai P., Tangchirapat W., Kalkornsurapranee E., Johns J., Nakaramontri Y. (2022). Utilization of high and low calcium oxide fly ashes as the alternative fillers for natural rubber composites: A waste to wealth approach. Ind. Crops Prod..

[23] Moonlek B., Saenboonruang K. (2019). Mechanical and electrical properties of radiation-vulcanized natural rubber latex with waste eggshell powder as bio-fillers. Radiat. Eff. Defects Solids..

[24] Hayichelaeh C., Boonkerd K. (2023). Utilization of palm oil as an alternative processing oil in carbon black-filled natural rubber compounds. Ind. Crops Prod..

[25] Sökmen S., Oßwald K., Reincke K., Ilisch S. (2021). Influence of Treated Distillate Aromatic Extract (TDAE) Content and Addition Time on Rubber-Filler Interactions in Silica Filled SBR/BR Blends. Polymers.

[26] Sergio R.-P., Susana R.-M., Alberto D.J., Socorro R.-M. (2019). *Leucaena leucocephala* extract has estrogenic and antiestrogenic actions on female rat reproduction. Physiol. Behav..

[27] Sereewatthanawut I., Prapintip S., Watchiraruji K., Goto M., Sasaki M., Shotipruk A. (2008). Extraction of protein and amino acids from deoiled rice bran by subcritical water hydrolysis. Bioresour. Technol..

[28] Rafi N.M., Halim N.R.A., Amin A.M., Sarbon N.M. (2015). Response surface optimization of enzymatic hydrolysis conditions of lead tree (*Leucaena leucocephala*) seed hydrolysate. Int. Food Res. J..

[29] Balderas-León I., Baigts-Allende D., Cardador-Martínez A. (2021). Antioxidant, angiotensin-converting enzyme, and α-amylase inhibitory activities of protein hydrolysates of Leucaena leucocephala seeds. CYTA J. Food..

[30] Lowry O., Rosebrough N., Farr A.L., Randall R. (1951). Protein measurement with the folin phenol reagent. J. Biol. Chem..

[31] (2018). Rubber—Measurement of Vulcanization Characteristics Using Curemeters—Part 3: Rotorless Curemeter.

[32] (2018). Rubber, Vulcanized or Thermoplastic—Determination of Hardness—Part 4: Indentation Hardness by Durometer Method (Shore Hardness).

[33] (2017). Rubber, Vulcanized or Thermoplastic—Determination of Tensile Stress-Strain Properties.

[34] Gbassi G., Yolou F., Sarr S., Atheba P., Amin C., Ake M. (2012). Whey proteins analysis in aqueous medium and in artificial gastric and intestinal fluids. Int. J. Biol. Chem. Sci..

[35] Vilas Dhumal C., Pal K., Sarkar P. (2019). Synthesis, characterization, and antimicrobial efficacy of composite films from guar gum/sago starch/whey protein isolate loaded with carvacrol, citral and carvacrol-citral mixture. J. Mater. Sci. Mater. Med..

[36] Barakat A., Gaillard C., Steyer J.-P., Carrere H. (2014). Anaerobic Biodegradation of Cellulose–Xylan–Lignin Nanocomposites as Model Assemblies of Lignocellulosic Biomass. Waste Biomass Valoriz..

[37] Reddy N., Yang Y. (2005). Biofibers from agricultural byproducts for industrial applications. Trends Biotechnol..

[38] Boopasiri S., Sae-Oui P., Siriwong C. (2022). Fabrication of microcrystalline cellulose/zinc oxide hybrid composite by hydrothermal synthesis and its application in rubber compounding. J. Appl. Polym. Sci..

[39] Younis U., Rahi A.A., Danish S., Ali M.A., Ahmed N., Datta R., Fahad S., Holatko J., Hammerschmiedt T., Brtnicky M. (2021). Fourier Transform Infrared Spectroscopy vibrational bands study of Spinacia oleracea and Trigonella corniculata under biochar amendment in naturally contaminated soil. PLoS ONE.

[40] Silverstein R.M., Webster F.X., Kiemle D. (2005). Spectrometric Identification of Organic Compounds.

[41] Manimaran P., Saravanakumar S.S., Mithun N.K., Senthamaraikannan P. (2016). Physicochemical properties of new cellulosic fibers from the bark of Acacia arabica. Int. J. Polym. Anal..

[42] Azmi M.A., Mokhtar N., Shoparwe N.F., Shukor H. (2021). Biosorption of CU(II) Ions by Leucaena Leucocephala Leave from Aqueous Solution. IOP Conf. Ser. Earth Environ. Sci..

[43] Md Salim R., Asik J., Sarjadi M.S. (2021). Chemical functional groups of extractives, cellulose and lignin extracted from native Leucaena leucocephala bark. Wood Sci. Technol..

[44] Kebelmann K., Hornung A., Karsten U., Griffiths G. (2013). Intermediate pyrolysis and product identification by TGA and Py-GC/MS of green microalgae and their extracted protein and lipid components. Biomass Bioenergy.

[45] Jagadeesh D., Jeevan Prasad Reddy D., Varada Rajulu A. (2011). Preparation and Properties of Biodegradable Films from Wheat Protein Isolate. J. Polym. Environ..

[46] Francisca Gómez-Rico M., Font R., Fullana A., Martín-Gullón I. (2005). Thermogravimetric study of different sewage sludges and their relationship with the nitrogen content. J. Anal. Appl. Pyrolysis.

[47] Schaberg A., Wroblowski R., Goertz R. (2018). Comparative study of the thermal decomposition behaviour of different amino acids and peptides. J. Phys. Conf. Ser..

[48] Ishiwatari M., Ishiwatari R., Sakashita H., Tatsumi T. (1995). Thermogravimetry and pyrolysis-GC of chlorophyll-a: With a special emphasis on thermal behavior of its phytyl chain. J. Anal. Appl. Pyrolysis.

[49] Moghadam M., Salami M., Mohammadian M., Delphi L., Sepehri H., Emam-Djomeh Z., Moosavi-Movahedi A.A. (2020). Walnut protein–curcumin complexes: Fabrication, structural characterization, antioxidant properties, and in vitro anticancer activity. J. Food Meas. Charact..

[50] De Angelis A., Gasco L., Parisi G., Danieli P.P. (2021). A Multipurpose Leguminous Plant for the Mediterranean Countries: Leucaena leucocephala as an Alternative Protein Source: A Review. Animals.

[51] Zhan Y.-H., Wei Y.-C., Tian J.-j., Gao Y.-Y., Luo M.-C., Liao S. (2020). Effect of protein on the thermogenesis performance of natural rubber matrix. Sci. Rep..

[52] Lhamo D., McMahan C. (2017). Effect of protein addition on properties of guayule natural rubber. Rubber Chem. Technol..

[53] Liu X.-X., He M.-F., Luo M.-C., Wei Y.-C., Liao S. (2022). The role of natural rubber endogenous proteins in promoting the formation of vulcanization networks. e-Polymers.

[54] Smitthipong W., Tantatherdtam R., Rungsanthien K., Suwanruji P., Klanarong S., Radabutra S., Thanawan S., Vallat M.F., Nardin M., Mougin K. (2014). Effect of Non-Rubber Components on Properties of Sulphur Crosslinked Natural Rubbers. Adv. Mat. Res..

[55] Wei Y.C., Liu G.X., Zhang L., Zhao F. (2022). Exploring the unique characteristics of natural rubber induced by coordination interaction between proteins and Zn^2+^. Polymer.

[56] Coran A.Y. (1964). Vulcanization. Part VI. A model and treatment for scorch delay kinetics. Rubber Chem. Technol..

[57] Qiao H.-Q., Gao W.-J., Xu C., Liu Y.-X., Chen X.-L., Xu H.-T., Ning Y.-H., Li Y.-J., Liu Y.-C. (2008). The interaction of Chlorophyll a with ZnO Nanoparticles Synthesized by Sol-gel Method. Chin. J. Lumin..

[58] Akahori Y., Kawahara S. (2023). Effect of water on the accelerated sulfur vulcanization of natural rubber. Polym. Test..

[59] Butler J., Freakley P.K. (1992). Effect of Humidity and Water Content on the Cure Behavior of a Natural-Rubber Accelerated Sulfur Compound. Rubber Chem. Technol..

